# Epigenetic Reprogramming of Cell Identity in the Rat Primary Neuron–Glia Cultures Involves Histone Serotonylation

**DOI:** 10.3390/cells14120905

**Published:** 2025-06-15

**Authors:** Anastasia A. Borodinova, Yulia A. Leontovich, Alexander P. Beletskiy, Alexander V. Revishchin, Galina V. Pavlova, Pavel M. Balaban

**Affiliations:** 1 Laboratory of Cellular Neurobiology of Learning, Institute of Higher Nervous Activity and Neurophysiology, Russian Academy of Sciences, Moscow 117485, Russia; 2 Laboratory of Neurogenetics and Genetics of Development, Institute of Higher Nervous Activity and Neurophysiology, Russian Academy of Sciences, Moscow 117485, Russia

**Keywords:** epigenetics, HDAC, histone serotonylation, neuron, glia, transcriptional program, cell identity, reprogramming

## Abstract

Epigenetic rearrangements can create a favorable environment for the intrinsic plasticity of brain cells, leading to cellular reprogramming into virtually any cell type through the induction of cell-specific transcriptional programs. In this study, we assessed how chromatin remodeling induced by broad-spectrum HDAC inhibitors affects cellular differentiation trajectories in rat primary neuron–glia cultures using a combination of transcriptomics, qPCR, and cytochemistry. We described the epigenetic regulation of transcriptional programs controlled by master transcription factors and neurotrophins in the context of neuronal and glial differentiation and evaluated the expression of representative cell-specific markers. The results obtained suggest that HDAC inhibitors reduce the proliferative potential of cultured cells and induce transcriptomic changes associated with cell differentiation and specialization. Particularly, we revealed a significant upregulation of genes typically expressed in neuromodulatory neurons and the downregulation of genes expressed in glia and inhibitory neurons. Transcriptional changes were accompanied by continuous elevation of histone serotonylation levels in both neurons and glia. Emerging shortly after HDAC inhibition, a complex chromatin remodeling, which includes histone serotonylation, persists over many hours in distinct brain cells. We assume that this sustained epigenetic mechanism likely helps to maintain transcriptional changes associated with cell fate commitment, possibly priming cells for long-term fate conversion.

## 1. Introduction

The mammalian brain exhibits remarkable cellular diversity generated through tightly regulated multistep differentiation of neural progenitor cells into specialized cells of various lineages (neurons, astrocytes, and oligodendrocytes) throughout brain development and later in adulthood. This process is governed by dynamic epigenetic rearrangements that establish lineage-specific gene expression programs [1]. Histone acetylation, which is controlled by the balance between histone acetyltransferase and histone deacetylase (HDAC) activities, plays a pivotal role in cell fate decisions. Multiple studies demonstrated that HDACs act as molecular switches that suppress or promote the differentiation of various brain cells depending on the developmental stage and cellular context. For instance, developing glial cells (oligodendrocytes and astrocytes) exhibit lower histone acetylation levels as compared to neural progenitors and immature neurons [2], reflecting their repressed neurogenic potential. Presumably, HDACs play an important role in this process by silencing neuron-specific genes in nonneuronal cells [3]. Numerous studies have examined the role of specific HDACs in the regulation of glial and neuronal fates in the course of cell differentiation [4,5,6,7,8]. At early developmental stages, corresponding to a high rate of differentiation of multipotent neural stem cells (NSCs), members of class I HDACs (HDAC1-3) appear to act redundantly to negatively regulate oligodendrocyte differentiation, which is rescued by inactivation of either HDAC1, HDAC2, or HDAC3 [4,7]. It has been proposed that HDAC1 transcriptionally controls the genes responsible for neurogenic programs [9], and its deletion affects the neuronal differentiation of multipotent NSCs [10]. In contrast, HDAC3 removal initiated neuronal differentiation of multipotent NSCs [7], while HDAC3 overexpression selectively stimulated differentiation of multipotent NSCs to astrocytes [4]. At a later stage of development, the regulatory role of HDACs is reversed. Thus, class I HDACs restricted the lineage commitment of the oligodendrocyte precursor cells (OPCs) and induced the oligodendrocyte differentiation both in vitro and in vivo [5,8]. Moreover, both HDAC1 and HDAC2 were necessary for neuronal differentiation of neural progenitor cells in vivo (NPCs) [11]. It has been noticed that HDAC3 acts bi-directionally as a molecular switch for oligodendrocytes and astrocytes fate decision: its cooperation with p300 histone acetyltransferase stimulates oligodendrocytes differentiation, while selective blockade of HDAC3 facilitates astrocytes lineage specification in OPCs [8]. Therefore, these data imply a changing role of HDACs during brain cell differentiation, which requires a specific time window for proper regulation of cell identity.

Considerable efforts were made to investigate the role of epigenetics in cell differentiation using the broad-spectrum HDAC inhibitors. It was found that HDAC inhibitors can trigger developmental plasticity in primary OPC cultures: their application prevents oligodendrocyte differentiation and astrocyte fate commitment [6] and activates proneural genes that revert OPCs into multipotent neural stem cells [12]. This effect may probably induce subsequent reprogramming into other cell types, but it requires further investigation. Application of the HDAC inhibitors in vitro and in vivo enhances the neuronal differentiation of progenitor cells, accompanied by an increased expression of different neuronal transcription factors [2,13,14]. HDACs have been found to modulate not only cell differentiation but also neuron specialization. Inhibition of HDAC activity negatively regulated the expression of genes responsible for GABA synthesis and the development of GABAergic inhibitory neurons in cortical neuron cultures [15]. On the contrary, inhibition of HDACs in organotypic raphe slice cultures stimulated the expression of genes responsible for serotonin synthesis and, through the AMPAR-CaMKII signaling cascade, enhanced its release [16]. 

Along with canonical histone modifications (e.g., acetylation and methylation), various non-canonical histone modifications, such as serotonylation, play a role in regulating cell differentiation [17]. The recently described covalent attachment of serotonin to histone H3 glutamine residues, called histone serotonylation, reveals a novel epigenetic mechanism linking serotonin signaling to brain cell differentiation and function [17,18,19,20]. However, the broader implications of serotonergic regulation in brain cell fate determination remain to be explored. 

Epigenetic rearrangements create a favorable environment for the intrinsic plasticity of brain cells [21] where they are able to transform into cells of a different lineage (transdifferentiation or direct reprogramming) or to return to a pluripotent state (reprogramming), from where they can differentiate into virtually any cell type. Transdifferentiation has been observed in natural conditions, including both pathological and normal developmental states. For instance, transdifferentiation occurs as a regenerative response to injuries: following brain injury, the mature striatal astrocytes are capable to transdifferentiate into functional cholinergic or GABAergic neurons [22]; similarly, after a spinal cord injury, the oligodendrocyte precursor cells, also known as NG2 glia, can generate excitatory and inhibitory propriospinal neurons [23]. In both cases, the newly generated neurons successfully integrate into existing neural networks. The oligodendrocyte progenitors and reactive astrocytes represent the most frequent targets for cellular reprogramming due to their higher lineage plasticity compared to other brain cells [24,25,26,27]. Conversion of glia into the subtype-specific neurons can be induced both in vitro and in vivo under certain experimental conditions aimed at changing the expression of proneural genes by transcription factors [28,29], microRNAs [30,31] and small-molecule cocktails [32,33,34]. Further unraveling of the molecular pathways underlying brain cell identity may offer new strategies for regenerative medicine and novel therapeutic approaches to repair or replace malfunctioning cells in neurological diseases such as Alzheimer’s disease or epilepsy [29,35,36].

This study addresses the epigenetic regulation of the intrinsic plasticity of various brain cells during differentiation. By applying broad-spectrum HDAC inhibitors to rat primary cultures, we examined how chromatin remodeling influences cell proliferation and differentiation using transcriptomics, qPCR, click-chemistry, and immunocytochemistry. We performed a complex characterization of the epigenetically regulated neuronal and glial transcriptional programs from the viewpoint of cell differentiation and evaluated the expression of several cell-specific markers. HDAC inhibitors were found to attenuate cell proliferation while promoting the expression of various master transcription factors involved in cell differentiation and specialization. The number of DAPI+ cells remained unchanged after treatment, but some cells apparently underwent qualitative changes in cellular phenotype. Particularly, we revealed a significant upregulation of genes normally expressed in neuromodulatory neurons (serotonergic, dopaminergic, and cholinergic), and the downregulation of genes expressed in glial cells and inhibitory neurons. These transcriptional changes coincided with elevated histone serotonylation levels observed in both neuronal and glial populations. These findings suggest a previously unrecognized connection between HDAC activity, serotonin signaling, and cell differentiation. We propose that the observed canonical and non-canonical histone modifications cooperate to reshape cellular identity, providing novel insights into potential molecular mechanisms driving cellular reprogramming for brain repair and regenerative therapies.

## 2. Materials and Methods

### 2.1. Animals

The experiments were carried out in newborn (P0–P1) Wistar rats (Pushchino breeding facility, Russia). All experimental procedures were conducted in accordance with the European Communities Council Directive of 24 November 1986 (86/609/EEC) on the protection of animals used for scientific purposes. The study protocol was approved by the Ethics Committee of the Institute of Higher Nervous Activity and Neurophysiology of RAS (ethical approval №03, 3 June 2021).

### 2.2. Rat Primary Cortical Neuron Cultures

Cell cultures were prepared as previously described [37]. For qPCR experiments, approximately 0.25–0.3 million isolated cells were plated into individual wells coated with poly-D-lysine hydrobromide (Sigma-Aldrich, Saint Louis, MO, USA). For ICC experiments, the same number of cells was placed in individual wells on 12 mm glass coverslips coated with poly-D-lysine hydrobromide. Primary cortical neuron cultures were grown for two weeks in a CO_2_ incubator (5% CO_2_, 37 °C). The culture medium was partially refreshed every 2–3 days. In all experiments, HDAC inhibitors were applied on the 14th day in vitro (DIV) without washout. Each experimental culture was processed in parallel with the appropriate time-matched control culture. To exclude possible intergroup variability, each “control”/“experiment” pair was grown in the wells of the same plate and processed in parallel. To take into account possible intragroup variability, cultures from different biological replicates were taken into the experiment.

### 2.3. Drugs 

For the experiments, we used two broad-spectrum histone deacetylase HDAC inhibitors with different chemical structures whose efficacy lies in different working ranges according to the manufacturers’ instructions and literature data. Numerous studies have demonstrated that TSA works in the nanomolar range [6,12,38,39], making it much more effective than sodium butyrate (NaB), which works in the millimolar range [6,40]. The working concentrations of TSA and NaB were chosen according to the literature and were based on our own pilot experiments preceding the previous publication [37], and they were effective enough to provide HDAC inhibition and suppress cell proliferation but insufficient to induce cell death [41,42]. The HDAC inhibitor trichostatin A (TSA, 100 nM, Sigma-Aldrich, Saint Louis, MO, USA) was applied for 19 h as previously described [37]. The HDAC inhibitor sodium butyrate (NaB, 5 mM, Sigma-Aldrich, Saint Louis, MO, USA) was applied for a longer time, 24 h.

### 2.4. Cell Proliferation Assay

Proliferating cells were identified by labeling replicated DNA with ethynyl deoxyuridine (EdU, Lumiprobe, Moscow, Russia). Following its incorporation into DNA, EdU was subsequently detected with a fluorescent azide (Alexa Fluor™ 488 Azide, Thermo Fisher Scientific, Eugene, OR, USA) via “click“ chemistry reaction.

EdU (10 μM) was added to half of the culture medium for 1 h before drug administration. After incubation with EdU, the medium was replaced with another half of the medium supplemented with the HDAC inhibitor TSA. After 19 h of incubation with TSA, cultures were fixed for 10 min at room temperature (RT) in 4% PFA and permeabilized for 15 min with 0.1% Triton X-100 in PBS. EdU-labeled cells were then stained for 30 min with a “click” reaction mixture (4 mM copper(II)-BTTAA complex, 100 mM ascorbic acid, and 12 μM dye azide in 100 mM Tris buffer, pH 8.5).

### 2.5. RNA Extraction and Sequencing 

Total RNA from primary neuron cultures was isolated using the ExtractRNA kit according to the manufacturer’s protocol (Evrogen, Moscow, Russia). The following library preparation and sequencing were performed at the Genomed company (Moscow, Russia). Briefly, mRNA was purified from a total RNA mixture using oligo(dT)-coated magnetic beads, followed by mRNA fragmentation and cDNA synthesis. The synthesized cDNA was then subjected to end repair, 3′-adenylation, and adapter ligation. Following amplification of cDNA fragments, the PCR products were purified using Agencourt® AMPure® XP Beads (Beckman Coulter, Brea, CA, USA) and dissolved in EB solution. The library was validated on the Agilent Technologies 2100 bioanalyzer (Santa Clara, CA, USA). The double-stranded PCR products were then heat-denatured and circularized using a splint oligo sequence to generate a final library of single-stranded circular DNA molecules. The library was amplified using phi29 DNA polymerase to create a DNA nanoball containing more than 300 copies of a single DNA molecule. Paired-end sequencing of the library was performed on the DNBseq-G400 platform at the Genomed company (Moscow, Russia).

### 2.6. RNA Sequence Alignment and Analysis 

High-quality reads were mapped onto the reference rat genome (Rnor6, ENSEMBL database) using STAR [43] with default parameters and raw read counts obtained using FeatureCounts [44]. To account for differences in library sizes and to allow comparison between samples, raw expression values were normalized using the “median-of-ratios” method and subjected to differential expression analysis using the R-package DESeq2 [45] with a chosen *p*-adjusted significance level of <0.05.

### 2.7. Visualization and Functional Annotation of DEGs

To generate heat maps, normalized gene expression values were additionally log-transformed using the regularized logarithm (Rlog) function from the DESeq2 package [45]. Log-transformed data are shown as z-score values in the graphs. Gene ontology enrichment of differentially expressed genes was performed using the Metascape web resource, version 3.5 [46]. Gene expression visualizations were carried out using the R packages ggplot2, ComplexHeatmap, and VennDiagram. 

### 2.8. qPCR Analysis

Verification of sequencing data was performed using quantitative real-time PCR (qPCR). An equal amount of total RNA from each sample (approximately 800 ng) was treated with DNaseI (Thermo Scientific, Waltham, MA, USA) and then taken for the first-strand cDNA synthesis using MMLV RT kit (Evrogen, Moscow, Russia) and random decamer primers (Evrogen). To verify sequencing data, we performed qPCR using SYBR-green mastermix reagent (Evrogen) and specific primer pairs (Supplemental Table S2). For each sample, the reaction was run in triplicates in 384-well plates on the CFX384 Touch Real-Time PCR (Bio-Rad, Hercules, CA, USA) according to the following cycling conditions: 95 °C for 5 min, 42 cycles of 95 °C for 30 s, 61 °C (60 °C for *Htr3a*) for 30 s, and 72 °C for 30 s. Values were normalized to the rat *Hprt* housekeeping gene (Supplemental Table S2). Relative mRNA expression was calculated with the standard ΔΔCt method. 

qPCR data are presented as mean ± s.e.m. The statistical significance of the differences between the groups was calculated with the Mann–Whitney U test due to the non-normal distributions and small sample size. Significance was set at *p* < 0.05. All qPCR experiments were performed in at least four biological replicates.

### 2.9. Immunocytochemistry (ICC)

Control and TSA-treated cultures of primary rat cortical neurons were fixed for 10 min with 4% paraformaldehyde, washed with PBS, and then permeabilized for 15 min with 0.1% Triton X100. Depending on the secondary antibodies used, the cells were blocked in 5% normal goat serum (NGS) or in 5% donkey serum dissolved in PBS for 1 h at room temperature. Then, cells were incubated with primary antibodies (Supplemental Table S3) for 1 h at room temperature and washed three times for 10 min with 0.05% Tween20 in PBS. Secondary antibodies were used under the same conditions. DAPI (1:1000) was applied for 10 min and then washed with PBS. Coverslips were mounted with self-made Mowiol mounting medium supplemented with 8% DABCO. When the dilution of antibodies ranged from 1:50 to 1:200, we performed immunostaining on the parafilm in a small volume of 30 mkl by inverting the coverslips with the cells facing down onto the antibody drop, covering with a lid to prevent air drying, and incubating for 1 h at room temperature. The coverslips were then returned to the plate and processed as usual. Quantitative ICC experiments were performed in at least three biological replicates.

### 2.10. Microscopy and Analysis

Microscopy was carried out using equipment of the Research Resource Center of IHNA and NPh RAS for functional brain mapping. Stained cell cultures were visualized using a fluorescence microscope (HS All-in-one Fluorescence Microscope BZ-9000, Keyence, Itasca, IL, USA) with 20×/0.75 numerical aperture (NA) or 60×/1.40 NA (oil-immersion) Plan Apo λ objectives (Nikon, Melville, NY, USA). Z-stack images were acquired using the BZ-II Viewer with identical exposure parameters applied separately to each channel for each pair of control and experimental coverslips. Subsequent image preparation and analysis were performed using ImageJ software version 1.53t (NIH). Densitometric analysis involved manually selecting stained cells using the ROI Manager tool and creating a set of ROIs for each fluorescence channel, followed by measuring the fluorescence intensity in a single image from the z-stack images and normalizing to the background fluorescence intensity averaged over five areas. Data are presented as median ± SD in % of control values. The statistical significance of the differences between the groups was calculated with the Mann–Whitney U test. Significance was set at *p* < 0.05.

## 3. Results

### 3.1. HDAC Inhibitors Shift the Gene Expression Profiles in Primary Neuron Cultures from Proliferation to Differentiation and Affect the Expression of Master Transcription Factors

**Figure 1:**
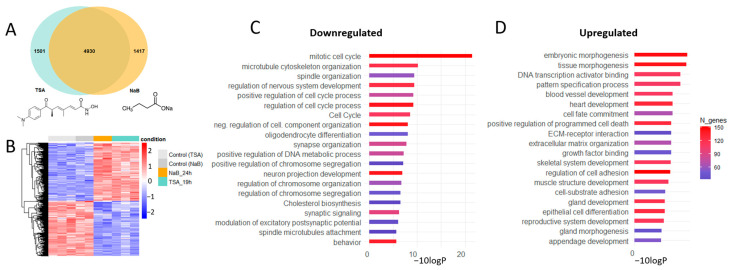
HDAC inhibitors TSA and NaB induce transcriptional changes in primary neuron cultures. (**A**)—Venn diagram illustrating the number of unique and overlapping differentially expressed genes (DEGs) in TSA-treated and NaB-treated neuron cultures. (**B**)—Cluster analysis of overlapping DEGs. Heatmap of 4930 overlapping DEGs showing differential expression in NaB-treated (orange) and TSA-treated neuron cultures (turquoise) compared to time-matched control cultures (gray). Values are shown as z-scored log-transformed normalized expression counts. The color scale indicates the expression levels (blue, low expression; red, high expression), measured in standard deviations from the row (genewise) mean. (**C**,**D**)—Gene Ontology (GO) analysis of the main enriched genes after HDAC inhibitors treatment. Bar charts show the top 20 most enriched GO terms for downregulated (**C**) and upregulated DEGs (**D**) based on Metascape. Gene ontologies are ranked by their significance.

To evaluate how epigenetic rearrangements affect gene expression profiles in brain cells, we performed bulk RNA sequencing of samples from rat primary neuron cultures treated with the broad-spectrum histone deacetylase (HDAC) inhibitor trichostatin A (TSA, 100 nM), as previously described [37]. In addition, to exclude possible non-specific actions of TSA on gene expression profile, we tested another broad-spectrum HDAC inhibitor, sodium butyrate (NaB, 5 mM), applied for 24 h. The transcriptome analysis revealed significantly overlapping datasets of 6431 and 6347 differentially expressed genes (DEGs) in cortical neuron cultures treated with TSA or NaB, respectively (Figure 1A) (Supplemental Table S1). Heatmap clustering of 4930 overlapping DEGs revealed a similar transcriptional profile for TSA-treated and NaB-treated cultures that differed from controls (Figure 1B). A significant number of the overlapping DEGs in the tested groups suggests that these HDAC inhibitors with different chemical structures share common regulatory pathways directly related to the regulation of transcriptional programs in primary neuron cultures.

**Figure 2:**
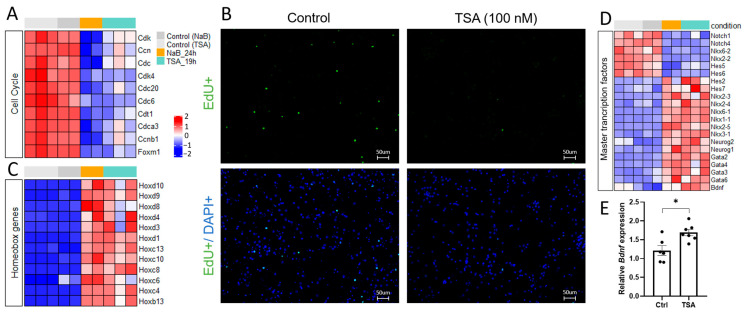
HDAC inhibitors impair cell proliferation and stimulate cell differentiation in rat primary neuron cultures. (**A**)—Heatmap illustrating representative downregulated genes associated with the cell cycle in NaB-treated (orange) and TSA-treated neuron cultures (turquoise) compared to time-matched control cultures (gray). Values are shown as z-scored log-transformed normalized expression counts. The color scale indicates the expression levels (blue, low expression; red, high expression), measured in standard deviations from the row (genewise) mean. (**B**)—Representative micrographs of EdU-positive proliferating cells (green) in control and TSA-treated primary neuron cultures, revealed by click-chemistry. Cell nuclei are labeled with DAPI (blue). The scale bar was 50 µM. Magnification was 20×. (**C**,**D**)—Heatmaps illustrating representative upregulated homeobox genes (**C**) and master transcription factors (**D**) that control embryonic morphogenesis and brain cell differentiation. The color scale indicates the expression levels (blue, low expression; red, high expression). (**E**)—Quantification of the relative mRNA expression of differentiation factor BDNF using qPCR. * *p* < 0.05.

Overlapping DEGs were represented by 2401 downregulated and 2529 upregulated genes (Supplemental Table S1). Gene Ontology (GO) analysis of DEGs datasets using Metascape, version 3.5 [46] revealed that a significant part of downregulated genes is involved in biological processes related to cell organization and proliferation (Figure 1C), while upregulated genes were engaged in cell differentiation and specialization and tissue and embryonic morphogenesis (Figure 1D). The clusters of downregulated genes were associated with cell division and DNA replication, including different cyclins and cyclin-dependent kinases (Figure 2A). To verify the influence of HDAC inhibitors on cell proliferation, we pretreated cell cultures for 1 h with 10 μM EdU, which incorporates into the newly synthesized DNA of proliferating cells. Subsequent analysis revealed a significant reduction in the number of EdU-positive cells in primary neuron cultures treated with TSA, reflecting a decrease in the proliferation rate (Figure 2B).

In the DEGs datasets, we found distinct classes of both downregulated and upregulated master transcription factors that work together to control embryonic morphogenesis, brain cell differentiation, and specialization (Figure 2C,D). Among the downregulated genes, we identified several transcription factors that control glial cell differentiation (Nkx6.2, Nkx2.2, Notch1, etc.) [26,47] (Figure 2D). In a dataset of upregulated genes, we identified proneural transcription factors from different families of homeobox proteins (Hox and Nkx), basic helix–loop–helix proteins (Neurog1 and Neurog2), zinc finger DNA binding proteins (Gata), and neurotrophins (Bdnf and NT4) (Figure 2C,D), known evolutionarily conserved master regulators of embryonic morphogenesis that form the neurogenic axis of cellular differentiation and promote neuron specialization [48,49,50,51,52,53]. Using qPCR, we confirmed that mRNA expression of brain-derived neurotrophic factor BDNF, the inducer of brain development and plasticity, was significantly upregulated in response to application of HDAC inhibitors (Figure 2E).

### 3.2. HDAC Inhibitors Regulate the Expression of Specific Markers of Various Brain Cells 

**Figure 3:**
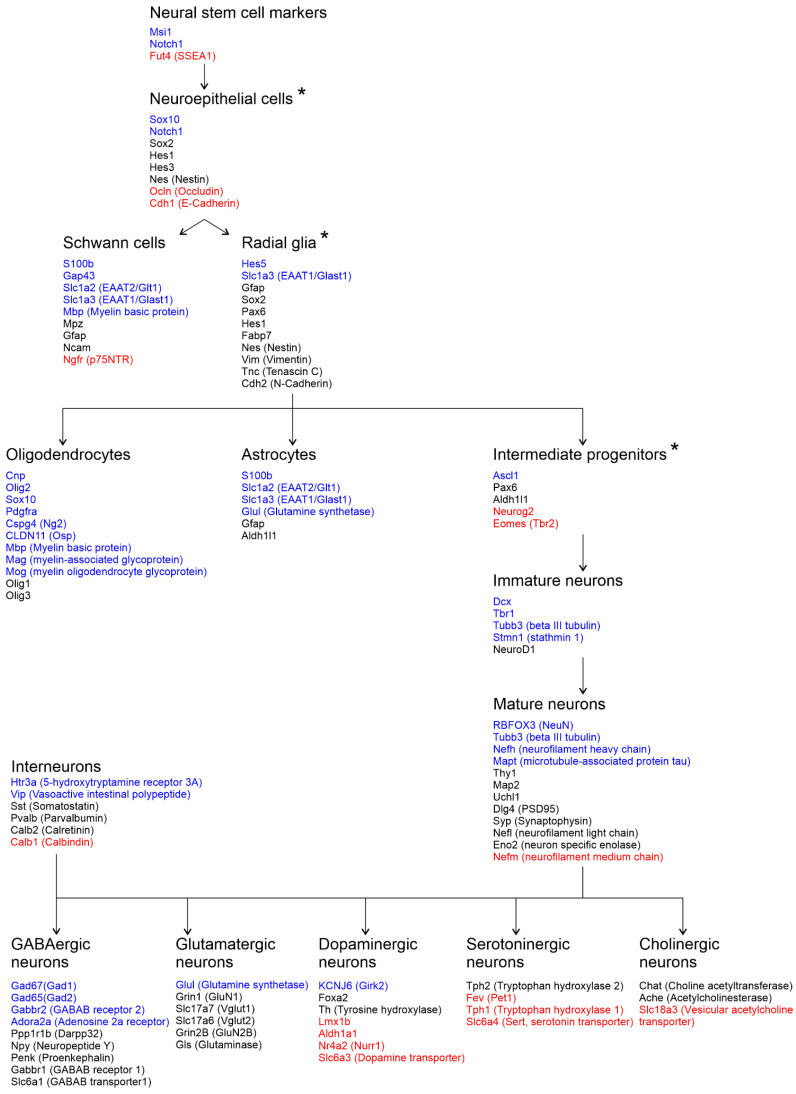
Scheme showing specific markers of diverse brain cells at different stages of their differentiation and specialization. Genes whose expression increases (red) or decreases (blue) after treatment of rat primary neuron cultures with histone deacetylase inhibitors are marked on the diagram. * labels the proliferating cells.

Given that treatment with HDAC inhibitors suppresses cell proliferation and regulates the expression of specific master transcription factors involved in cell differentiation, we sought to characterize clusters of epigenetically controlled genes associated with differentiation and specialization of individual brain cells. In a diagram on Figure 3, we have combined publicly available data on the generally accepted unique markers of differentiating and mature brain cells (https://www.cellsignal.com/pathways/neuronal-and-glial-cell-markers, accessed on 22 April 2024; https://www.abcam.com/neuroscience/neural-markers-guide, accessed on 22 April 2024) with the results of our transcriptome analysis of downregulated (blue) and upregulated (red) differentially expressed genes (DEGs) from overlapping datasets (Figure 3). Several cell-specific markers in the diagram were subsequently used for downstream analyses.

**Figure 4:**
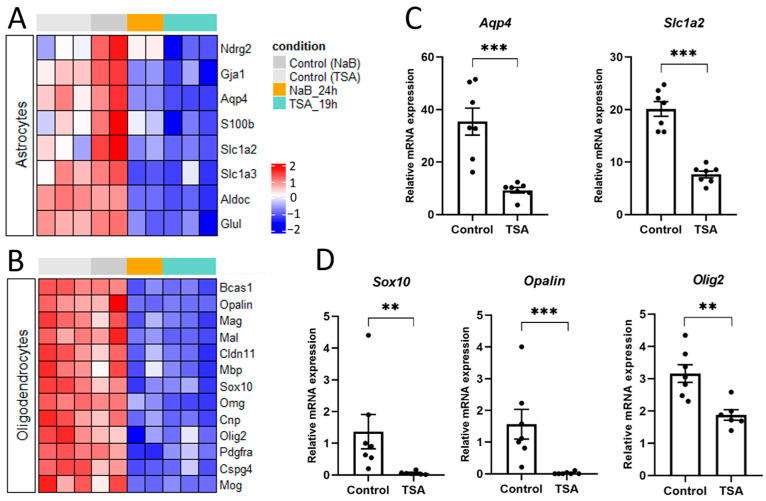
HDAC inhibitors alter mRNA expression of specific glial cell markers. (**A**,**B**)—Heatmaps illustrating representative downregulated genes encoding specific astrocyte markers (**A**) and oligodendrocyte markers related to the myelin synthesis process (**B**) in NaB-treated (orange) and TSA-treated neuron cultures (turquoise) compared to time-matched control cultures (gray). Values are shown as z-scored log-transformed normalized expression counts. The color scale indicates the expression levels (blue, low expression; red, high expression), measured in standard deviations from the row (genewise) mean. (**C**,**D**)—qPCR verification of RNAseq data using specific primer pairs for astrocyte (**C**) and oligodendrocyte markers (**D**). ** *p* < 0.01, *** *p* < 0.001.

We found that transcription of multiple glial and neuronal markers was affected in rat primary neuron cultures in response to application of the HDAC inhibitors NaB or TSA. Particularly, the transcriptome analysis revealed a profound loss of unique glial markers typical for astrocytes (Figure 4A), as well as for oligodendrocytes (OL) and oligodendrocyte precursor cells (Figure 4B). For the following analysis, we selected several genes (*Aqp4*, *Slc1a2* aka EAAT2, or GLT1) encoding the membrane protein aquaporin 4 and the excitatory amino acid transporter EAAT2, which are primarily expressed in astrocytes and play a critical role in their function [54]. The selected OL genes (*Sox10*, *Opalin* aka Tmem10, and *Olig2*) encode the transmembrane protein opalin, involved in OL lineage commitment, and its downstream transcriptional factors Sox10 and Olig2, required for OL differentiation [55]. The RNA sequencing data were confirmed by quantitative PCR with pairs of primers for selected astrocyte (Figure 4C) and oligodendrocyte markers (Figure 4D). 

Our results are consistent with previously published data, showing the requirement of HDACs for oligodendrocyte differentiation, while less is known about their involvement in astrocyte differentiation [4,5,6,7,8].

**Figure 5:**
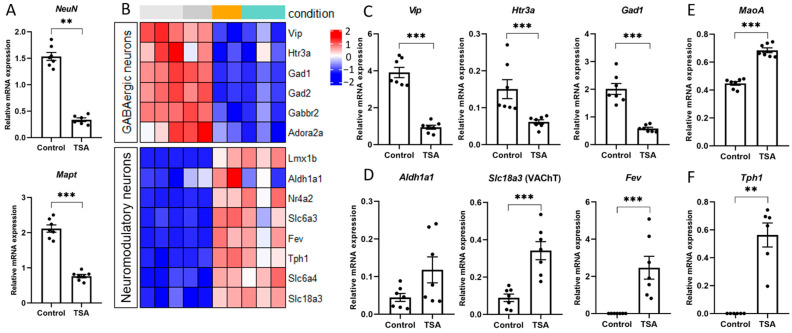
HDAC inhibitors influence mRNA expression of neuronal markers. (**A**)—qPCR verification of RNAseq data using specific primer pairs for neuronal markers. (**B**)—Heatmap illustrating representative downregulated and upregulated genes specific for GABAergic interneurons (**top panel**) and neuromodulatory neurons (**bottom panel**) in NaB-treated (orange) and TSA-treated neuron cultures (turquoise) compared to time-matched control cultures (gray). Values are shown as z-scored log-transformed normalized expression counts. The color scale indicates the expression levels (blue, low expression; red, high expression), measured in standard deviations from the row (genewise) mean. (**C**)—qPCR verification of RNAseq data using specific primer pairs for *Htr3a*, *Vip*, and *Gad1* genes specific for GABAergic interneurons. (**D**)—qPCR verification of RNAseq data using specific primer pairs for genes, specific for distinct neuromodulatory neurons, including *Aldh1a1* (dopaminergic), *Slc18a3* (cholinergic), and *Fev* (serotonergic). (**E**,**F**)—qPCR analysis of the expression of *Tph1* and *MaoA* genes encoding crucial enzymes for serotonin metabolism. ** *p* < 0.01, *** *p* < 0.001.

The mRNA expression of key neuron-specific markers, such as the pro-neural transcription factor NeuN (Rbfox3) and the cytoskeletal proteins Tubb3 (beta III tubulin) and Mapt (microtubule-associated protein tau) [56], was affected in our experimental conditions based on transcriptome analysis (Figure 3) and qPCR (Figure 5A). Interestingly, with a general decline in the expression of neuronal markers, the transcription of specific markers of different neuronal subpopulations was regulated in opposite ways (Figure 5B). Similarly to previous data [15], we found that the expression of interneuron markers, associated with GABA synthesis, transport, and signaling (*Gad1*, *Gad2*, *Gabbr2,* and *Slc6a11*), was significantly downregulated after the application of HDAC inhibitors (Figure 5B,C). Since the expression of *Htr3a* and *Vip* genes was decreased upon induction of epigenetic rearrangements (Figure 5C), we hypothesized that the most profound transcriptional changes affected the Htr3a+/VIP+ interneuron population, which constitutes approximately one-third of cortical interneurons [57].

Interestingly, HDAC inhibitors positively regulate the expression of genes associated with secretory phenotypes of neuromodulatory neurons [16,58]. Indeed, in our experiments on primary neuron cultures, the expression of genes encoding transcription factors (*Fev*, *Lmx1b*, and *Nr4a2*), enzymes (*Tph1*, *Aldh1a1*, and *MaoA*), and transporters (*Slc6a4*, *Slc6a3*, and *Slc18a3*), specific for distinct neuromodulatory neurons, was significantly upregulated in response to epigenetic rearrangements induced by the HDAC inhibitors (Figure 3 and Figure 5B). For the following analysis, we selected several markers specifically expressed in cholinergic neurons (*Slc18a3* aka VAChT) and in subpopulations of dopaminergic neurons (*Aldh1a1*) [59]. The group of selected serotonergic genes (*Fev* aka Pet1, *Tph1,* and *MaoA*) included the transcription factor Pet1, which controls the differentiation and function of serotonergic neurons [60,61], and the key enzymes Tph1 and MaoA, responsible for the 5-HT turnover. Using qPCR, we confirmed that the application of HDAC inhibitors stimulated the expression of serotonergic and cholinergic genes but did not influence the expression of dopaminergic genes (Figure 5D–F). Presumably, this effect is directly related to the regulation of gene transcription. However, the known indirect neuroprotective effects of HDAC inhibitors on the survival of neuromodulatory neurons and the maintenance of their phenotype should be noted [62].

Thus, our results in primary neuron cultures, while consistent with previously published data, additionally demonstrate multiple parallel trajectories of differentiation/de-differentiation processes simultaneously driven by epigenetic rearrangements. 

### 3.3. HDAC Inhibitor Trichostatin A Elevates the Expression of Genes Associated with Serotonergic Secretory Phenotype and Stimulates Histone Serotonylation

**Figure 6:**
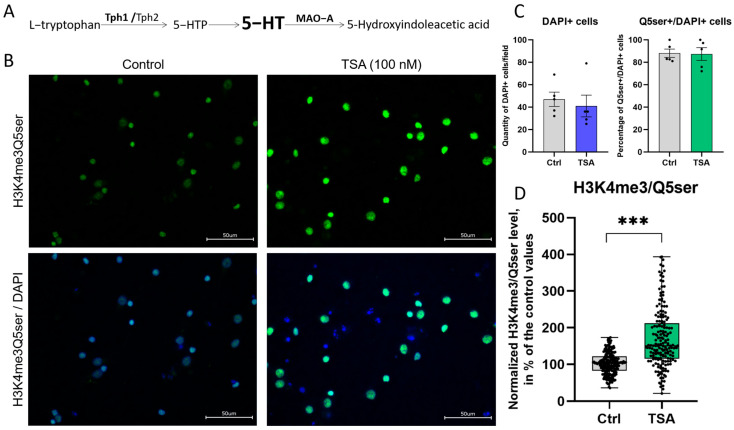
HDAC inhibitor trichostatin A (TSA) enhances histone serotonylation in rat primary cortical cultures. (**A**)—Scheme of serotonin (5-HT) synthesis and metabolism with indication of key enzymes. (**B**)—Fluorescent microscopy of cell cultures stained with antibodies against H3K4me3Q5ser that targets histone H3 trimethylated on lysine 4 and serotonylated on glutamine 5 (green) and DAPI (blue). The scale bar was 50 µM. Magnification was 60×. (**C**)—Quantification of DAPI+ cells per field and percentage of H3K4me3Q5ser+ cells relative to the total number of DAPI+ cells in control and TSA-treated cultures. Results are presented as mean ± s.e.m. (**D**)—The box plot shows the results of densitometric analysis of histone serotonylation levels in control and TSA-treated cultures. Data were normalized and calculated as a % of control values in each biological replicate; each point shows the value for the individual cell, n = 5. Results are presented as median ± SD. *** *p* < 0.001.

During detailed analysis of the upregulated DEGs dataset, we revealed a subset of genes encoding important components of the serotonergic cells’ functioning, including the 5-HT transporters (*Slc6a4* aka Sert and *Slc18a1* aka VMAT1) and enzymes for the synthesis (*Tph1*) and degradation (*Maoa*) of 5-HT. To verify the RNA sequencing data, we performed qPCR with specific primer pairs for the *Tph1* (Figure 5F) and *Maoa* (Figure 5E) genes, encoding key enzymes responsible for the 5-HT turnover (Figure 6A), and confirmed that the application of TSA significantly increases the expression of target genes in both neurons and glia (Supplemental Figure S1A). These results are consistent with previously published data on serotonergic neurons and non-neuronal cells that describe HDACs as negative regulators of serotonergic phenotype and demonstrate positive influence of their inhibitors on the expression of different genes involved in serotonin turnover [16,63,64]. In these studies, the activation of serotonin-associated genes was accompanied by the accumulation, release, and uptake of 5-HT in cultured cells. Potentially, similar effects could be observed in our experimental conditions.

Given that the 5-HT synthesis enzyme Tph1 can be expressed in non-neuronal cells, we examined its distribution both in neurons and in glia in primary cultures. Our preliminary results confirmed that Tph1 is widely expressed in differentiating cultured brain cells (Supplemental Figure S1C), providing them with the ability to synthesize serotonin. 

Next, we questioned what the functions of serotonin are in differentiating cell cultures. It is well known that 5-HT acts as a classical neurotransmitter, modulating synaptic processes, but it also acts as a factor involved in cell differentiation [65]. Recent studies suggest that some effects may occur through histone serotonylation, the posttranslational histone modification, when serotonin covalently binds to glutamine residues on histone proteins by the endogenous tissue transglutaminase 2 (TGM2) and alters gene transcription through epigenetic rearrangements [17]. According to our transcriptomic data, *Tgm2* gene expression was predicted to be increased by the HDAC inhibitors. We hypothesized that, if true, this may contribute to the serotonylation of histones in primary cultures of cortical neurons treated with HDAC inhibitors. To test this hypothesis, cortical neuron cultures were stained with antibodies that recognize the previously described dual permissive mark H3K4me3Q5ser on histones that combine serotonylated glutamine at position 5 (Q5ser) on histone H3 and its neighboring trimethylated lysine at position 4 (H3K4me3) [17]. We observed widespread expression of the H3K4me3Q5ser mark in both control and TSA-treated primary neuron cultures, more than in 80% of all cells (Figure 6B,C). Taking into account that the percentage of DAPI+ cells expressing the histone serotonylation mark H3K4me3Q5ser was not changed (Figure 6C), a significant elevation of histone serotonylation in TSA-treated cells was confirmed by densitometric analysis of images (Figure 6D). We noticed that the total number of DAPI+ cells was unchanged (Figure 6C), which implies that the enhanced histone serotonylation was not due to changes in cellular quantity and composition. We conclude that HDAC inhibitors stimulate the expression of serotonergic genes in primary cultures of cortical neurons, which ensures the production of 5-HT and serotonylation of histones in the cells, potentially involved in the changes in cell differentiation trajectories.

### 3.4. Histone Serotonylation Marks Are Widely Distributed Across Different Populations of Both Neurons and Glia

**Figure 7:**
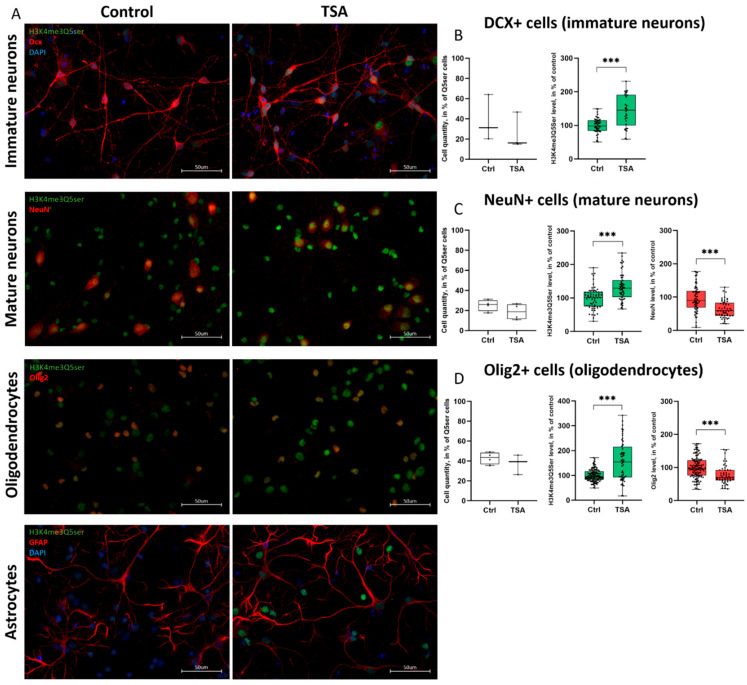
Various populations of brain cells were affected by the HDAC inhibitor trichostatin A (TSA). (**A**)—Fluorescent microscopy shows co-localization of H3K4me3Q5ser marks (green) with one of the cell-specific markers (red) and DAPI (blue). The scale bar was 50 µM. Magnification was 60×. (**B**–**D**)—Figures show the ratio of double-labeled cells relative to the total number of H3K4me3Q5ser+ cells (**left panel**), densitometric analysis of histone serotonylation levels (**middle panel**), and the levels of cell-specific transcription factors in control and TSA-treated cultures. Data were normalized and calculated as a % of control values; each point shows the value for the individual cell. Results are presented as median ± SD. *** *p* < 0.001.

Widespread expression of the H3K4me3Q5ser mark raises the question of which cell populations undergo changes in histone serotonylation following HDACs inhibition. Immunocytochemical analysis revealed substantial overlap of the H3K4me3Q5ser mark with the main neuronal (Dcx, NeuN) and glial markers (GFAP, Olig2) (Figure 7A). We quantified a percentage of H3K4me3Q5ser+ cells that express cell-specific markers in control and TSA-treated groups and found no differences (Figure 7B–D, left panel). However, TSA treatment caused a significant increase in histone serotonylation levels in all double-labeled cells tested, including immature Dcx^+^ and mature NeuN^+^ neurons, as well as Olig2-positive oligodendrocytes (Figure 7B–D, middle panel). These changes were accompanied by a significant decrease in the mRNA (Figure 4D and Figure 5A) and protein expression of transcription factors NeuN and Olig2 (Figure 7C,D, right panel), responsible for cell specialization.

Since the elevated levels of histone serotonylation were detected in both neurons and glial cells, we wondered whether this was a cause or a consequence of widespread transcriptional changes induced by HDAC inhibitors. The time-course experiments showed early downregulation of gene expression (*Olig2* and *Sox10*) (Supplemental Figure S2A) along with increased histone serotonylation in cell cultures (Supplemental Figure S2B). The results obtained indicate that transcriptional changes were accompanied by increased histone serotonylation, which appeared after 4 h of incubation with TSA and persisted for at least 19 h. Given that histone serotonylation was increasing in distinct brain cells along with changing expression of cell-specific transcription factors, we speculate that chromatin remodeling induced by histone serotonylation contributes to the changes in a transcriptional program associated with cell fate commitment and cell specialization, but it requires further investigation.

## 4. Discussion

Studying the principles of regulation of cell fate commitment is important for understanding both normal neurodevelopment and the pathological mechanisms underlying neurodegenerative diseases, where cellular identity is often disrupted. Epigenetic modifiers of the chromatin landscape play a crucial role in the regulation of transcriptional programs essential for the establishment and maintenance of cellular phenotype. Changes in the activity of various epigenetic modifiers influence chromatin compaction and DNA accessibility to transcription factors, creating a favorable environment for the intrinsic plasticity of brain cells [21]. 

In the current study, we aimed to determine how chromatin rearrangements, induced by broad-spectrum HDAC inhibitors, affect cellular differentiation trajectories. Unlike previous studies on specialized cell lines or multipotent stem/progenitor cells, we used differentiating rat primary neuron–glia cultures to describe epigenetic regulation of transcriptional programs in distinct brain cells using RNA sequencing. To eliminate possible side effects and to increase the reproducibility and reliability of our results, we tested two different HDAC inhibitors with different chemical structures in parallel. 

### 4.1. HDAC Inhibition Suppresses Pro-Glial Transcriptional Programs and Promotes Neuronal Differentiation

Trichostatin A (TSA) and sodium butyrate (NaB) produced very similar transcriptional changes (Figure 1A,B, Supplemental Table S1) that include downregulation of genes involved in cell organization and proliferation (Figure 1C and Figure 2A), glial fate commitment, and glial differentiation (Figure 2D and Figure 4). The reduction in the proliferative potential of primary cultures was confirmed by click-chemistry with EdU, which incorporates into the newly synthesized DNA of proliferating cells (Figure 2B). These results are consistent with previous findings demonstrating that HDACs are required for cell proliferation and neurogenesis [66,67] while their inhibition with small molecules (TSA, NaB, and valproic acid) attenuates significantly cell proliferation, stimulates the expression of proneural transcription factors, and promotes neuronal differentiation both in vitro and in vivo [2,14].

In the overlapping DEGs datasets, we identified distinct classes of master transcription factors that work together to control cell differentiation and specialization (Supplemental Table S1). Particularly, the expression of transcription factors promoting glial cell differentiation (Olig2, Sox10; Nkx6.2, Nkx2.2, Notch1, etc.) [26,47] was significantly downregulated, as confirmed by transcriptome analysis (Figure 2D), qPCR (Figure 4), and ICC (Figure 7D). This was accompanied by a decrease in the expression of different glial markers, with the most pronounced suppression of oligodendrocyte genes (Figure 4B, Supplemental Table S1). HDAC activity was shown to be essential for glial differentiation and maturation. HDACs interact with regulatory regions on chromatin and control specific transcriptional programs [7]. We know from the literature that distinct glial cells (oligodendrocytes and astrocytes) lose open chromatin marks during differentiation as compared to multipotent cells and neurons [2,68]. In fact, HDACs associated with repressor complexes containing Sin3A or NcoR co-repressors [69] are recruited to regulatory regions on chromatin to control glial cell identity through the repression of proneural gene activity in non-neuronal cells [3,7]. Additionally, HDACs can promote the expression of pro-glial factors [7], likely through the previously described interaction with histone acetyltransferases [8].

Numerous studies have demonstrated that chromatin remodeling induced by HDAC inhibitors prevents neural progenitor cells (NPCs) differentiation into glial cells and favors differentiation into neurons [2,13,14,70]. In our transcriptomic data we observed an increased expression of proneural transcription factors from distinct families of homeobox proteins (Hox and Nkx), basic helix–loop–helix proteins (Neurog1 and Neurog2), zinc finger DNA binding proteins (Gata), and neurotrophic factors (Bdnf and NT4) (Figure 2D and Figure 3), which form the neurogenic axis of cell differentiation [53], determine neural lineage commitment, and promote neuronal differentiation and specialization [48,49,50,52]. Surprisingly, several markers of fully mature neurons (Mapt and NeuN) were significantly downregulated, as confirmed by qPCR and ICC staining (Figure 3, Figure 5A,B, and Figure 7C). Interestingly, some studies describe their heterogeneous expression in different subpopulations of neurons, as well as in glial cells [71,72,73]. Therefore, we suggest that downregulation of “neuronal” markers, observed in our experiments, is in fact a reflection of significantly altered transcriptional programs underlying different cellular identities, which may lead to changes in cell composition, possibly through initiation of cellular reprogramming.

### 4.2. HDAC Inhibition Mediates Neuron Specialization

Heterogeneous by nature, distinct neuron subpopulations have a certain set of properties that are acquired through a certain transcriptional program, controlled by specific transcription factors. These transcriptional programs can be epigenetically regulated in different ways during neuron specialization [15,16]. In our transcriptomic data, we found opposite regulation of genes specifically expressed in subpopulations of inhibitory neurons and neuromodulatory neurons. Particularly, we observed downregulation of multiple interneuron genes involved in GABA synthesis, transport, and signaling following HDAC inhibitor treatment (Figure 5B,C), as previously shown [15]. Recent data suggest that cortical interneurons are represented by three populations of cells selectively expressing either parvalbumin (PV), somatostatin (SST), or ionotropic serotonin receptor 5HT3a (Htr3a); the latter, in turn, is divided into VIP-positive and VIP-negative subpopulations [57]. We confirmed the downregulation of *Htr3a* and *Vip* genes encoding interneuron markers upon induction of epigenetic rearrangements (Figure 5C). Given that, we assume that a subset of cortical Htr3a^+^/VIP^+^ and/or Htr3a^+^/VIP^-^ interneurons were most likely affected, but this requires further investigation.

Interestingly, the downregulation of the GABAergic genes in primary neuron cultures was counterbalanced by the upregulation of multiple genes associated with neuromodulatory neuron phenotypes (Figure 3). Among the upregulated DEGs, we identified master transcription factors (Gata2, Gata3, Fev aka Pet1, Lmx1b, and Nr4a2 aka Nurr1) that are responsible for establishing and maintaining the phenotypes of serotonergic and dopaminergic neurons [49,52,74,75]. This was accompanied by the elevated expression of the corresponding cell-specific markers, particularly, the important components of serotonin turnover (Figure 5B,D–F). We confirmed that the serotonin synthesis enzyme Tph1 is widely expressed in cortical neurons and astrocytes of primary neuron–glia cultures (Supplemental Figure S1C), and its transcription is elevated by the HDAC inhibitors (Figure 5F, Supplemental Figure S1A), which may increase the ability of cells to synthesize serotonin. These results are in agreement with previous studies describing (1) the negative influence of HDACs on the serotonergic phenotype and (2) the positive influence of HDAC inhibitors on serotonergic gene expression, accompanied by increased serotonin synthesis and release [16,63,64].

### 4.3. HDAC off, Serotonin on: Serotonylation as a Novel Epigenetic Mechanism

It is well established that serotonin is a multifaceted regulator of many biological processes in the brain, acting in synapses as a neurotransmitter and in the nucleus as a chromatin remodeling factor. Serotonin serves as a donor of monoamine groups for posttranslational modification of various proteins, called serotonylation, catalyzed by tissue transglutaminase 2 (TGM2) [76]. Recent studies have demonstrated that TGM2 mediates serotonylation of glutamine residues on histone proteins [17,77,78]. One paper by Ballestar and colleagues reported that TGM2 is capable of modifying multiple glutamine residues in chicken erythrocyte core histones [77]. However, histone serotonylation in brain cells describes the only glutamine Q5 residues on histone H3 proteins as a validated target for TGM2, although it is unclear whether other positions on histone H3 and other histones can be serotonylated in different brain cells or conditions [17]. H3Q5 serotonylation has been shown to result in the stabilization of adjacent H3K4me3 marks and the creation of a dual permissive mark H3K4me3Q5ser, which facilitates gene expression programs essential for brain cell differentiation [17,20,79]. Given that serotonylation was only recently discovered, compared to other covalent protein modifications such as phosphorylation and methylation, very little is known about the possible target genes it affects, and only a few studies describe the functional consequences of histone serotonylation on brain development and function [17,18,19,20]. 

According to our transcriptomic data, expression of the *Tgm2* gene was upregulated after the application of HDAC inhibitors. We hypothesized that this might stimulate histone serotonylation under our experimental conditions. Indeed, immunocytochemical staining revealed increased levels of histone serotonylation in TSA-treated primary cortical cultures (Figure 6B,D). Elevation of histone serotonylation marks H3K4me3Q5ser was widely distributed across different populations of both neurons and glial cells, as confirmed by co-localization with various cellular markers (Figure 7). We found that the temporal dynamics of gene expression in TSA-treated cell cultures (Supplemental Figure S2A) were accompanied by increased histone serotonylation, which appeared after 4 h of incubation with TSA and persisted for at least 19 h (Supplemental Figure S2B; Figure 6B).

We hypothesized that the observed switching of transcriptional programs associated with phenotypically distinct brain cells indicates a reshaping of cell identity, possibly through the initiation of reprogramming processes. Previous studies support our hypothesis, demonstrating that induction of neuronal fate conversion both in vitro and in vivo can be achieved by (epi)genetic manipulations with a single transcription factor or a panel of transcription factors in glial cells (astrocytes and oligodendrocyte precursor cells) [12,24,28,29,80]. However, these data should be interpreted with caution, as transcriptional shifts are not necessarily sufficient for complete functional reprogramming of brain cells. Although some neuronal subpopulations were found to change their secretory phenotypes following genetic manipulation with transcription factors [81], the reprogramming efficiency and complete maturation of differentiated neurons depend on, but are not limited to, a complex permissive microenvironment created by chromatin remodeling and neurotrophic factors [24,69,82].

These data support our hypothesis that the observed complex transcriptional changes, involving a number of transcription factors and neurotrophins, most likely contributed to the onset of cellular reprogramming under our experimental conditions. We hypothesized that HDAC inhibitors induce chromatin remodeling by creating open chromatin marks that disrupt existing steric barriers to Tgm2 activity [83] and facilitate histone serotonylation in regulatory regions of target genes. This results in serotonylation of glutamine residues at position 5 (Q5ser) on histone H3 and stabilization of adjacent trimethylated lysine at position 4 (H3K4me3) by creating a dual permissive mark H3K4me3Q5ser that facilitates gene transcription through interaction with the transcription initiation factor TFIID [17]. Emerging shortly after HDAC inhibition, a complex chromatin remodeling, which includes enhanced histone acetylation [37] and histone serotonylation, persists over many hours in distinct brain cells. This sustained epigenetic mechanism likely helps to maintain transcriptional changes associated with cell fate commitment, possibly priming cells for long-term fate conversion. 

### 4.4. Limitations of the Study

- Bulk RNA sequencing results should be taken with caution because primary neuronal cultures consist of highly heterogeneous populations of various types of brain cells, likely at different stages of development. More precise assessment of gene expression programs in individual brain cells using scRNA-seq would be preferable.
- The incubation times for TSA and NaB were different. As a continuation of previous research [37], TSA was applied for 19 h. The sodium butyrate data with the appropriate time-matched controls were taken from a separate project where conditions were the same except for the longer incubation time (24 h), which was chosen as a more generally accepted value. These two datasets were taken to strengthen our conclusions and to avoid possible side effects caused by each drug. We believe that these differences in the incubation times for TSA and NaB were not relevant since the initial goal of long-term use of HDAC inhibitors was to assess which late-response genes were affected following chromatin rearrangements. We understand that some genes may be underestimated with this approach, but the overall gene expression pattern appears to be the same.
- Most of our conclusions regarding changes in transcriptional programs were based on RNA sequencing. Only a few transcriptional factors were confirmed by the ICC staining. We can only speculate that transcriptional changes may lead to changes in the proteome, but this requires additional verification.
- We speculate that induced histone modifications may trigger processes associated with cell identity regulation and cellular reprogramming. However, actual complete reprogramming of one cell into another cannot be confirmed in this experimental paradigm, as it requires a longer time, possibly several days/weeks.

### 4.5. Conclusions and Future Directions

Multiple studies have examined the role of HDACs in regulating neurogenesis, synaptic plasticity, and cognitive function in the normal, aging, or diseased brain [9,10,11,84,85]. The limited ability of the brain to recover from injury or resist pathological changes during neurodegeneration determines the need for HDAC inhibitors to stimulate regenerative processes and compensate for functional deficits [84,85,86,87]. 

This study highlights the critical role of HDAC-mediated chromatin remodeling as an epigenetic mechanism for the regulation of neuronal and glial transcriptional programs, shaping brain cell identity and plasticity. Our results support previous studies describing HDACs as key “molecular switches” whose activity (or inhibition) influences brain cell phenotypes, with important implications for both physiological adaptation and disease. By uncovering the interplay between HDACs, serotonin signaling, and brain cell differentiation trajectories, this work opens new avenues for understanding potential molecular mechanisms driving cellular reprogramming. Further investigation will require sequencing-based lineage tracing to identify reprogrammed cells, confirmation of their complete functional reprogramming, and assessment of their functional integration into existing ensembles using electrophysiological approaches. Future studies should leverage single-cell omics and in vivo models to translate these findings into targeted interventions for brain repair in neurodevelopmental and neurological disorders and regenerative therapies. 

## Data Availability

Original data are available upon reasonable request. The RNA sequencing datasets are available at the Gene Expression Omnibus GEO database with ID GSE297880.

## Supplementary Materials

The following supporting information can be downloaded at: https://www.mdpi.com/article/10.3390/cells14120905/s1, Table S1: contains lists of differentially expressed genes (DEGs) in rat primary cortical neuron cultures treated with either trichostatin A (TSA), or sodium butyrate (NaB), list of overlapping DEGs, GO terms for upregulated and downregulated DEGs from the overlapping dataset; Table S2: contains list of specific primer pairs used for qPCR analysis; Table S3: contains list of primary and secondary antibodies used for ICC staining; Figure S1: analysis of Tph1 expression in neuronal and glial cultures using qPCR and ICC; Figure S2: qPCR analysis of the temporal dynamics of transcription factor expression (4 h, 48 h), and ICC analysis of histone serotonylation levels (4 h) in control and TSA-treated primary neuron cultures; Figures S3–S5: raw results include validation of qPCR primer pairs using gel electrophoresis.
