# Supplementary material for: Epigenetic Reprogramming of Cell Identity in the Rat Primary Neuron–Glia Cultures Involves Histone Serotonylation
Source: Cells. 2025 Jun 15;14(12):905. doi: 10.3390/cells14120905 (PMC12191383; doi:10.3390/cells14120905)
Supplement: Supplementary file 1 [file cells-14-00905-s001.zip › Supplemental_information.pdf]

Supplemental Information

EPIGENETIC REPROGRAMMING OF CELL IDENTITY IN THE RAT PRIMARY NEURON-GLIA CULTURES INVOLVES HISTONE SEROTONYLATION

Borodinova A.A.[1*], Leontovich Yu.A.[1], Beletskiy A.P.[1], Revishchin A.V.[2], Pavlova G.V.[2], Balaban P.M.[1]

[1] Laboratory of Cellular Neurobiology of Learning, Institute of Higher Nervous Activity and Neurophysiology, Russian Academy of Sciences, Moscow, Russia.

[2] Laboratory of Neurogenetics and Genetics of Development, Institute of Higher Nervous Activity and Neurophysiology, Russian Academy of Sciences, Moscow, Russia.

* Correspondence: borodinova.msu@mail.ru

Supplemental_Table_S2. The list of specific primer pairs used for qPCR analysis.

**Type**	**Name**	**Efficiency**	**Sequence**
Housekeeping gene	*Hprt*	1.82	for, 5′- CGTCGTGATTAGTGATGATGAAC-3′; rev, 5′- CAAGTCTTTCAGTCCTGTCCATAA-3′;
Oligodendrocytes	*Sox10*	2	for, 5′- GCAGAAAGTTAGCCGACCA-3′; rev, 5′- GCCGAGGTTGGTACTTGTAG-3′;
	Opalin	1.74	for, 5′-GACTGTGGTCCGTCTGTTG-3′; rev, 5′-TCTTGGGATTGTCATAGAGTTCTG-3′;
	Olig2	1.84	for, 5′- ACTACTGATACGGGACTGGAT-3′; rev, 5′- GTTCTTCTGTCAAGCCCTCA-3′;
Astrocytes	*Aqp4*	1.95	for, 5′- GGCAGGTGCACTTTACGAGTATG-3′; rev, 5′- GCCAGTTCTAGGGAGTCTCTTCTG-3′;
	Slc1a2	1.93	for, 5′- AGGCCCCTGAAGAAACTAAGAT-3′; rev, 5′- CCAGCGGGGAATACCACA-3′;
Neurons	*NeuN*	1.96	for, 5′- CAACTTACGGAGCGGCACTGGAG-3′; rev, 5′-GACCCTGGAAGCAAACGGACAAGA-3′;
	Mapt	1.98	for, 5′-CCTCCGCTGTCCTCTTCTGTCCTC-3′; rev, 5′-GTCCTCCATGTTCGGGGTGTCTC-3′;
Monoamine neurons	*MaoA*	1.94	for, 5′-ATTAGTGCCATCCCACCGAT-3′; rev, 5′-TGGTTTAGTGTCATCCAGAGTGAT-3′;
Serotonergic neurons	*Fev*	*	for, 5′- AGCCCTGCGGTACAGAAAG-3′; rev, 5′- GGGTCGGTGAGCTTGAACT-3′;
	Tph1	*	for, 5′- TGGTTCCCAAAGAAGATTTCTGA-3′; rev, 5′- GTTTGTAGTTCATAGCCAGCTCT-3′;
Dopaminergic neurons	*Aldh1a1*	1.93	for, 5′- CTTTGCCAATGCGTACTTGTC-3′; rev, 5′- CCAATAGGTTCACGTCTCGTAA-3′;
Cholinergic neurons	*Slc18a3*	*	for, 5′- GCGCGATGTGTTGCTTGATG-3′; rev, 5′-GCTGCGGGAGTAATAGTTGTAGTC-3′;
Interneurons	*Gad1*	1.76	for, 5′- AGATCCTGGTTGACTGTAGAGAC-3′; rev, 5′-TGGTATTGGCAGTTGATGTCAG-3′;
	Htr3a	*	for, 5′- CAGTAACAGCTATGCGGAAATGAAG-3′; rev, 5′- AGCCATGCACACTACAAAGTAGA-3′;
	Vip	1.69	for, 5′- CAGAATGCCTTAGCGGAGAA-3′; rev, 5′- ATTCGTTTGCCAATGAGTGAC-3′;
Neurotrophins	*Bdnf*	1.88	for, 5′- CCATAAGGACGCGGACTTGTAC -3′; rev, 5′-AGACATGTTTGCGGCATCCAGG -3′;

*- The expression of the target gene is low in the analyzed samples. The specified primer pairs allow detection of the PCR product in quantities insufficient for serial dilution of the sample required for primer efficiency analysis. Relative mRNA expression was calculated with an efficiency of 2.

Supplemental_Table_S3. The list of primary and secondary antibodies used for ICC staining.

Description	Host	Target	Dilution	
Primary	Rabbit	Polyclonal anti-histone H3Kme3Q5Ser antibodies to serotonylated histones	1:500	ABE2580, Merck
	Rabbit	Polyclonal anti-Tph1 antibodies to tryptophan hydroxylase 1	1:400	DF6465, Affinity Biosciences
	Mouse	Monoclonal anti-NeuN antibodies to neuron-specific nuclear protein NeuN	1:500	MAB377, Merck
	Mouse	Monoclonal anti-GFAP antibodies to glial fibrillary acidic protein	1:500	custom made antibodies*
	Mouse	Monoclonal anti-beta III tubulin antibodies	1:50	ab7751, Abcam
	Goat	Polyclonal anti-doublecortin (DCX) antibodies	1:200	sc-8066, Santa Cruz
	Goat	Polyclonal anti-Olig2 antibodies to oligodendrocyte lineage-specific basic helix-loop-helix transcription factor Olig2	1:50	sc-19969, Santa Cruz
Secondary	Goat	Polyclonal anti-rabbit IgG (H+L), cross-adsorbed, Alexa488-conjugated antibodies	1:500	A11008, Invitrogen
	Goat	Polyclonal anti-mouse IgG (H+L), highly cross-adsorbed, Alexa594-conjugated antibodies	1:500	A11032, Invitrogen
	Donkey	Polyclonal anti-goat IgG (H+L), Alexa594-conjugated antibodies	1:50	705-585-147, Jackson Immunoresearch
	Donkey	Polyclonal anti-rabbit IgG (H+L), Cy2-conjugated antibodies	1:50	711-225-152, Jackson Immunoresearch
* The custom made primary monoclonal anti-GFAP antibodies was kindly gifted by Alexander Moshchenko (FCBRN FMBA, Russia).				

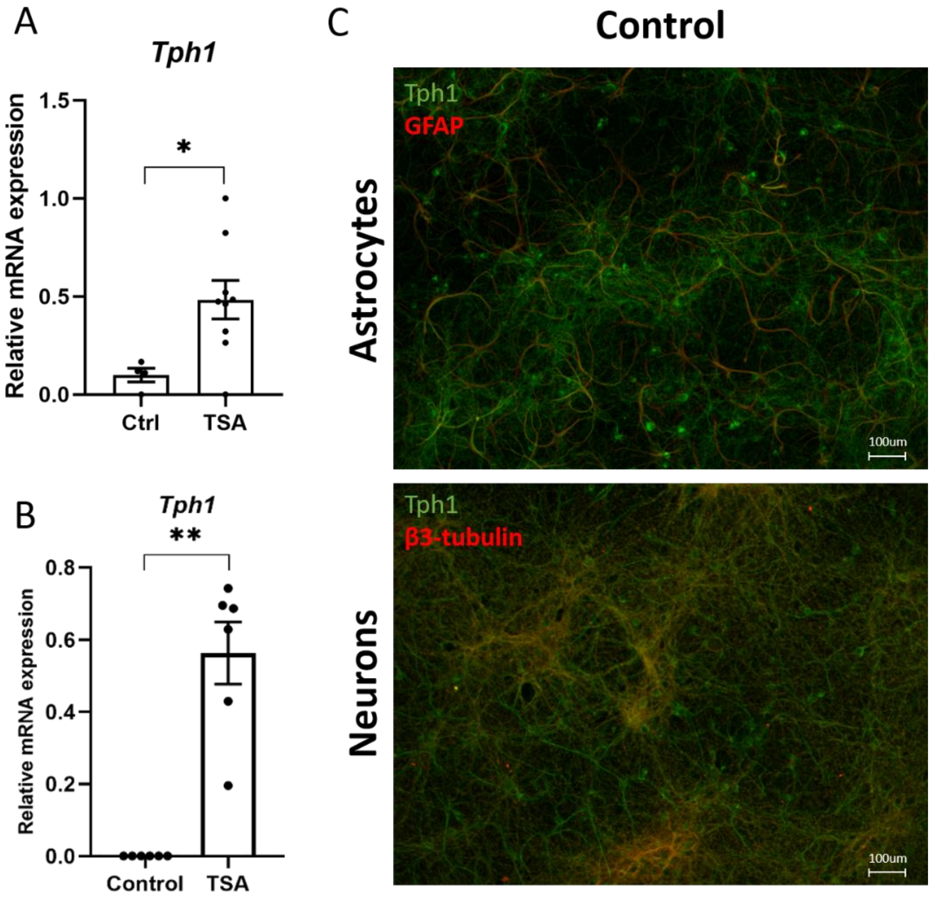

Supplemental_Fig_S1. The expression of 5-HT synthesis enzyme Tph1 in neurons and glia. A– qPCR analysis of mRNA expression of Tph1 in control and TSA-treated mixed glial cultures. B – qPCR analysis of mRNA expression of Tph1 in control and TSA-treated primary neuron cultures (The same as Fig.5F). Results are presented as Mean ± s.e.m., * $p < 0.05$, Mann-Whitney U test; C – Immunocytochemical staining of primary neuron cultures and analysis of Tph1 (green) co-localization with astrocyte marker GFAP (red, top panel) or neuron-specific marker β3-tubulin (red, bottom panel). Scale bar was 100 μM. Magnification 20x.

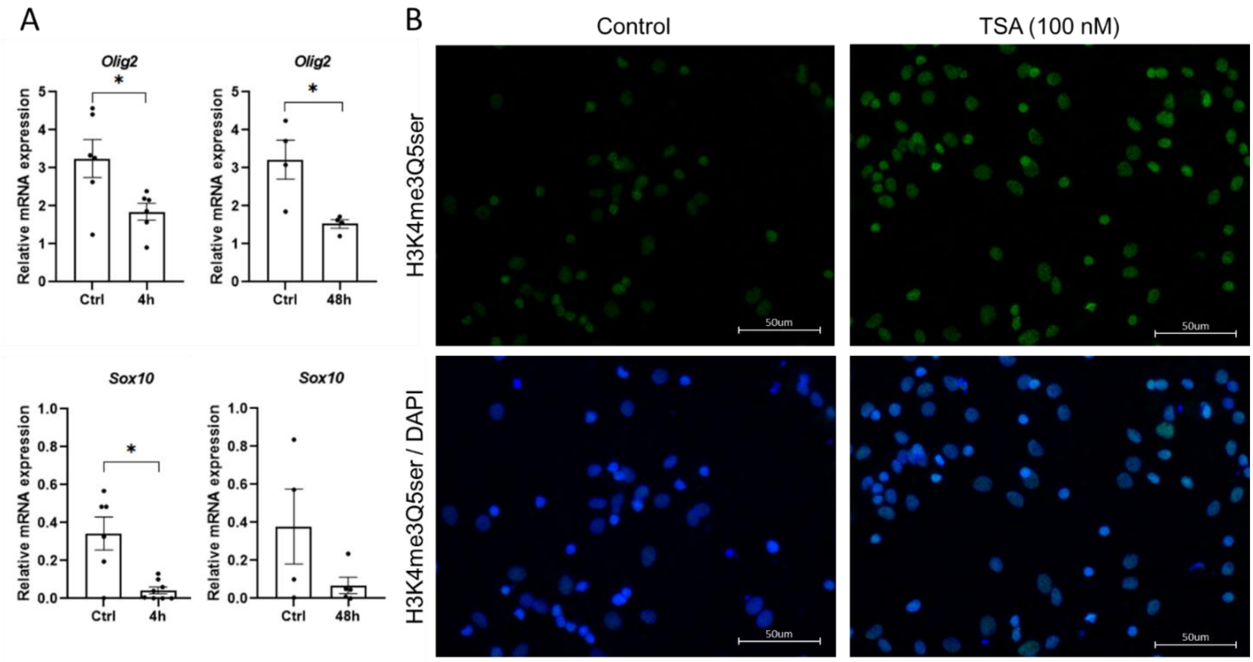

Supplemental_Fig_S2. HDAC inhibitor trichostatin A (TSA) induces early and persistent transcriptional changes accompanied by enhanced histone serotonylation. A –qPCR analysis of the temporal dynamics of mRNA expression of the cell-specific transcription factors Olig2 and Sox10 in control and TSA-treated primary neuron cultures. Results are presented as Mean ± s.e.m., * $p < 0.05$, Mann-Whitney U test. B – Fluorescent microscopy of representative control primary neuron cultures and cultures treated with TSA for 4 hours. Cultures were stained with antibodies against H3K4me3Q5ser that targets histone H3 trimethylated on lysine 4 and serotonylated on glutamine 5 (green), and DAPI (blue). Scale bar was 50 µM. Magnification 60x

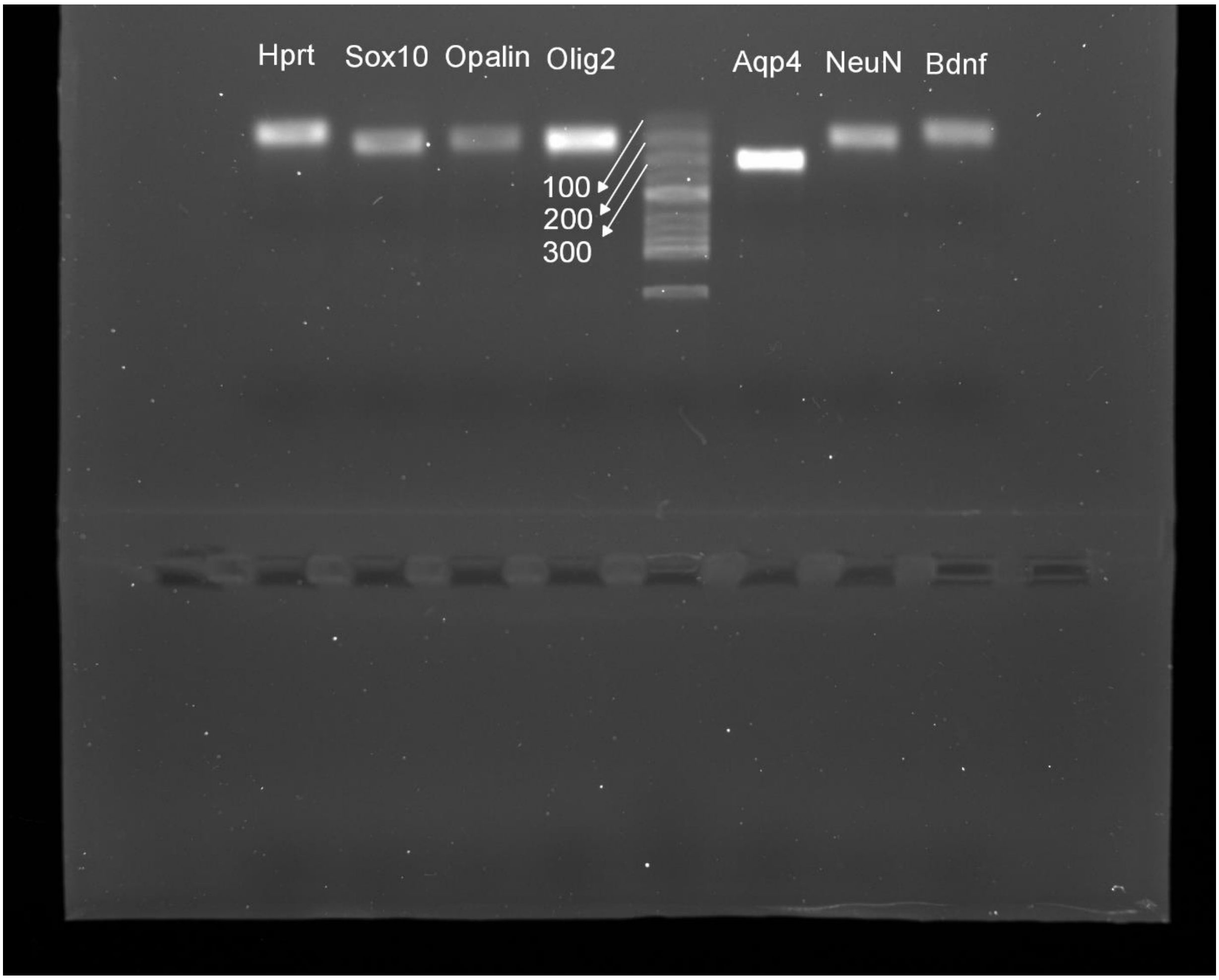

Supplemental_Fig_S3. Verification of primer pairs used for quantitative PCR in 1% agarose gel. PCR was run with the parameters described in the Methods section. For amplicon size analysis, a 100bp+ ladder (Evrogen, Moscow, Russia) was used.

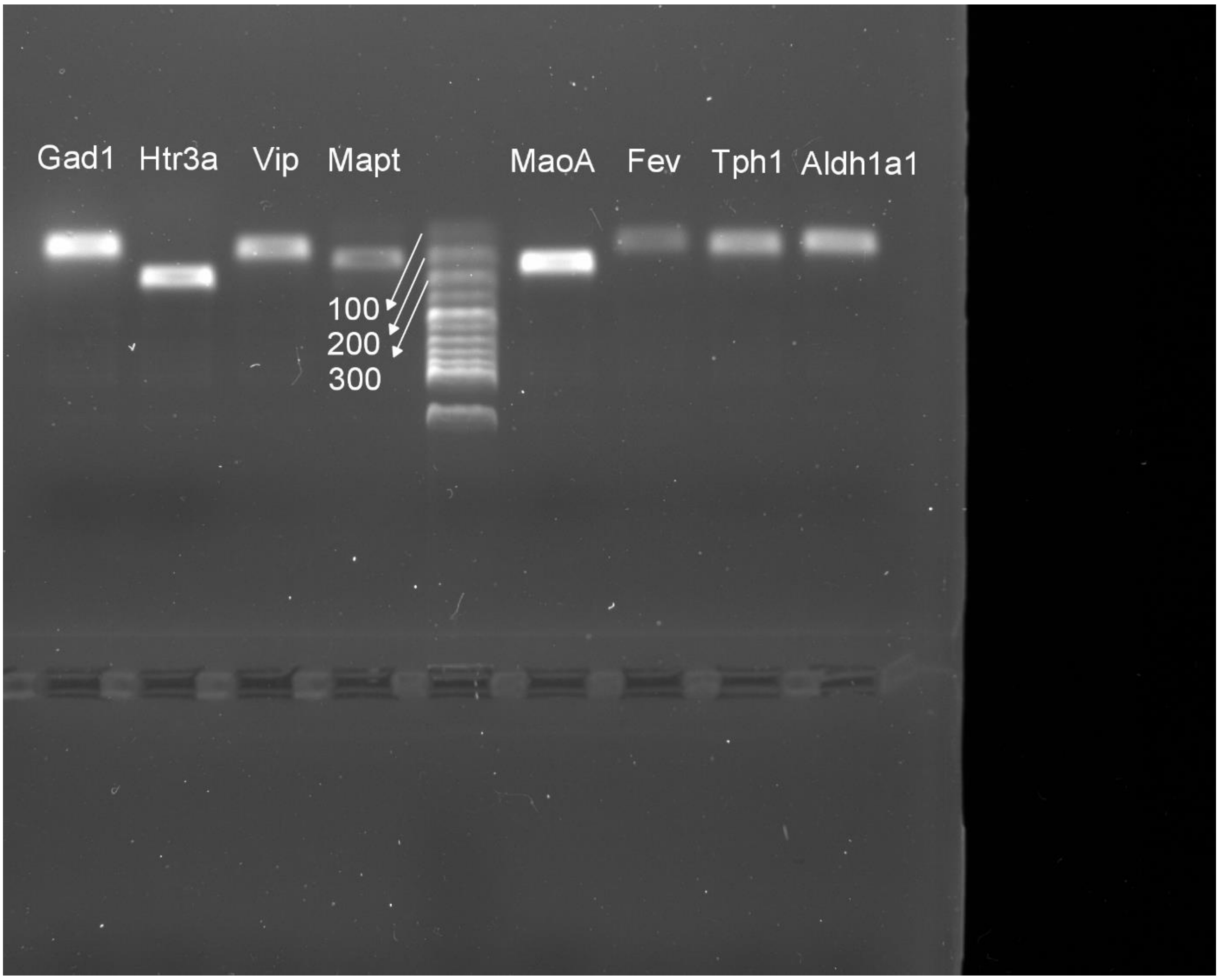

Supplemental_Fig_S4. Verification of primer pairs used for quantitative PCR in 1% agarose gel. PCR was run with the parameters described in the Methods section. For amplicon size analysis, a 100bp+ ladder (Evrogen, Moscow, Russia) was used.

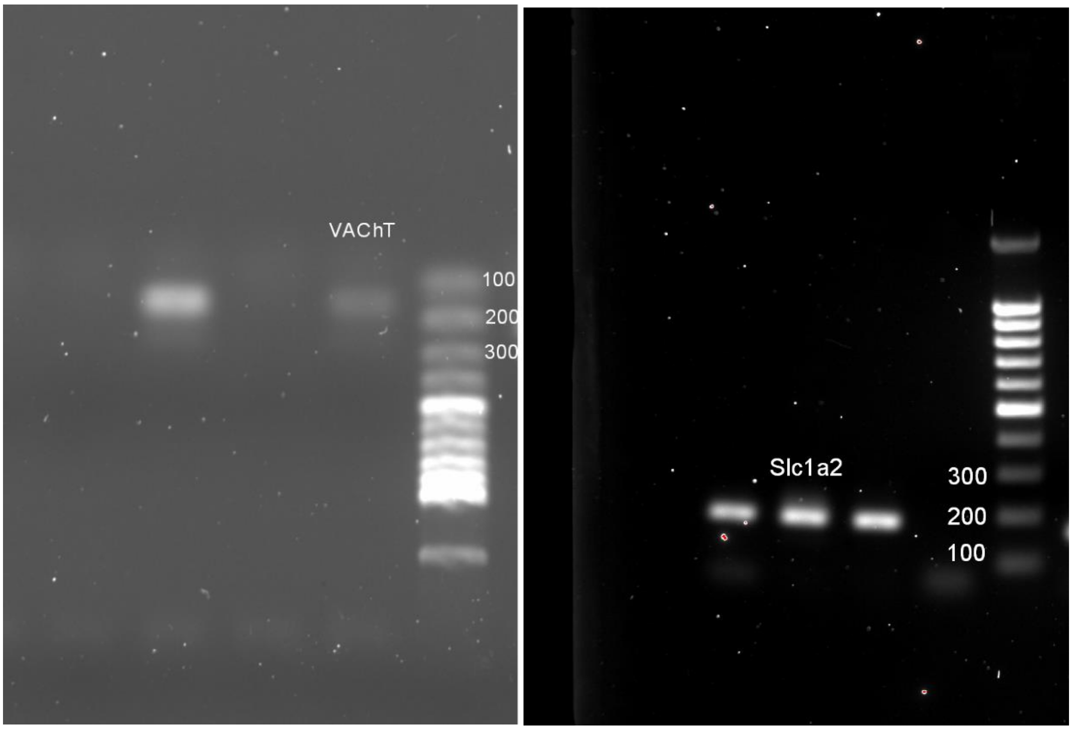

Supplemental_Fig_S5. Verification of primer pairs used for quantitative PCR in 1% agarose gel. PCR was run with the parameters described in the Methods section. For amplicon size analysis, a 100bp+ ladder (Evrogen, Moscow, Russia) was used.
